# TKTL1 Knockdown Impairs Hypoxia-Induced Glucose-6-phosphate Dehydrogenase and Glyceraldehyde-3-phosphate Dehydrogenase Overexpression

**DOI:** 10.3390/ijms23073574

**Published:** 2022-03-25

**Authors:** Inês Baptista, Effrosyni Karakitsou, Jean-Baptiste Cazier, Ulrich L. Günther, Silvia Marin, Marta Cascante

**Affiliations:** 1 Department of Biochemistry and Molecular Biomedicine, Faculty of Biology, Universitat de Barcelona (UB), 08028 Barcelona, Spain; inesvbaptista@gmail.com (I.B.); e.karakitsou@ub.edu (E.K.); 2 Institute of Biomedicine of University of Barcelona (IBUB), University of Barcelona (UB), 08028 Barcelona, Spain; 3 Institute of Cancer and Genomic Sciences, University of Birmingham, Edgbaston, Birmingham B15 2TT, UK; j.cazier@bham.ac.uk; 4 Centre for Computational Biology, University of Birmingham, Edgbaston, Birmingham B15 2TT, UK; 5 Institute of Chemistry and Metabolomics, University of Lübeck, 23562 Lübeck, Germany; ulrich.guenther@uni-luebeck.de; 6 CIBER of Hepatic and Digestive Diseases (CIBEREHD), Institute of Health Carlos III (ISCIII), 28029 Madrid, Spain

**Keywords:** metabolism, hypoxia, transketolase-like 1, pentose phosphate pathway, transcriptomics, leukemia, AML, glucose-6-phosphate dehydrogenase, glyceraldehyde-3-phosphate dehydrogenase

## Abstract

Increased expression of transketolase (TKT) and its isoform transketolase-like-1 (TKTL1) has been related to the malignant leukemia phenotype through promoting an increase in the non-oxidative branch of the pentose phosphate pathway (PPP). Recently, it has also been described that TKTL1 can have a role in survival under hypoxic conditions and in the acquisition of radio resistance. However, TKTL1’s role in triggering metabolic reprogramming under hypoxia in leukemia cells has never been characterized. Using THP-1 AML cells, and by combining metabolomics and transcriptomics techniques, we characterized the impact of TKTL1 knockdown on the metabolic reprogramming triggered by hypoxia. Results demonstrated that TKTL1 knockdown results in a decrease in TKT, glucose-6-phosphate dehydrogenase (G6PD) and glyceraldehyde-3-phosphate dehydrogenase (GAPDH) activities and impairs the hypoxia-induced overexpression of G6PD and GAPDH, all having significant impacts on the redox capacity of NADPH- and NADH-related cells. Moreover, TKTL1 knockdown impedes hypoxia-induced transcription of genes encoding key enzymes and transporters involved in glucose, PPP and amino acid metabolism, rendering cells unable to switch to enhanced glycolysis under hypoxia. Altogether, our results show that TKTL1 plays a key role in the metabolic adaptation to hypoxia in THP-1 AML cells through modulation of G6PD and GAPDH activities, both regulating glucose/glutamine consumption and the transcriptomic overexpression of key players of PPP, glucose and amino acids metabolism.

## 1. Introduction

Synthesis of ribose-5-phosphate (R5P) is crucial to ensure the production of nucleotide building blocks for DNA duplication. R5P can be synthesized through both branches of the pentose phosphate pathway (PPP), mediated by glucose-6-phosphate dehydrogenase (G6PD) on the oxidative side with the additional production of nicotinamide-adenine dinucleotide phosphate (NADPH) and controlled by TKT on the non-oxidative side, which links glycolysis to the production of R5P [1,2].

The TKTL1 isoenzyme is missing 38 amino acids in the active site [3] and increased expression of TKT and its isoform TKTL1 has been reported in different types of cancers [4,5,6,7], and is related to malignant transformation [8] and poor prognosis [9,10,11,12]. Evidence across several cancer tissues up until now have highlighted that TKTL1 is tightly linked to adaptation to hypoxia and its overexpression has been correlated with resistance to ionizing radiation and chemotherapy [13] as well as to the “Warburg effect” [14]. For example, in glioma cells, it has been demonstrated that hypoxia led to TKTL1 induction and that its knockdown promoted cell death [13]. Induction of TKTL1 under hypoxia has also been observed in different cell lines of colorectal cancer (CRC) and upregulation has been demonstrated for a subset of CRC patients [10]. It has also been reported that hypoxia induced major metabolic changes in AML cell lines, including adaptation of both lipid and glycolytic metabolism [15]. In addition, increased levels of TKTL1 have been reported in response to decitabine (DAC) treatment in primary acute myeloid leukemia (AML) samples [16] and correlated to the acquisition of tyrosine kinase inhibitor resistance in chronic myeloid leukemia (CML) [17].

However, to date, there has been little research regarding the importance of TKTL1 for the metabolic response triggered by hypoxia in leukemia and its specific role during metabolic reprogramming of AML cells towards hypoxia remains unknown.

One of the objectives of this work is to untangle the functional relationship between TKTL1 expression and metabolic adaptation of AML cells to hypoxia. For this purpose, we performed a comparative analysis of hypoxia-induced changes using the THP1 cell line, a monocytic leukemic cell line established in 1980 from the blood of an AML child patient with an *MLL* fusion [18]. Using THP1 transduced with a lentiviral vector expressing a TKTL1-specific shRNA (TKTL1^KD^) and a wild-type control transduced with an empty vector (THP-1^WT^), we attested the differences between the two cell lines at the transcriptional and metabolic levels. Together, our findings highlight that in response to hypoxia, THP-1 cells deeply rewire central metabolism at the transcriptomic level to sustain an enhanced consumption of glucose and glutamine, and that this response is severely impaired in the cells lacking TKTL1 that are not able to increase the activity of key players in redox homeostasis such as GAPDH and G6PD.

## 2. Results

### 2.1. TKTL1 Contributes to Cell Growth and Pentose Phosphate Pathway in Normoxia and Hypoxia

**Figure 1:**
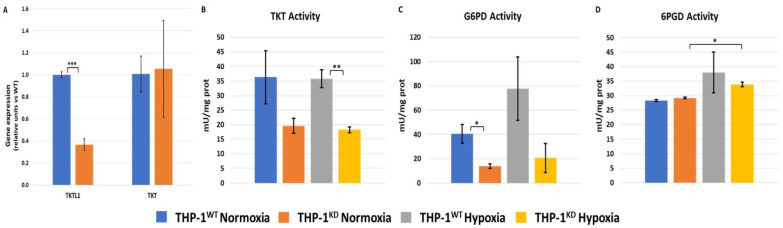
Effects of TKTL1 knockdown on TKTL1 and TKT gene expression and the pentose phosphate pathway. (**A**) Effects of TKTL1 silencing on TKTL1 and TKT gene expression. RT-PCR performed using PPA1 as a housekeeping gene for data normalization. Decrease in TKTL1 expression determined as 63.3% ± 0.03. Data shown as mean ± SD (n = 3) of normalized values. Enzymatic activity assays performed through spectrophotometry in normoxia and hypoxia (1% O_2_); for (**B**) transketolase (TKT), (**C**) glucose-6-phosphate dehydrogenase (G6PD) and (**D**) 6-phospo-gluconate dehydrogenase (6PGD). Data represented as mean ± SD (n = 2). Statistically significant differences in all panels were determined by two-tailed independent sample Student’s *t*-test: *p* < 0.05 (*). *p* < 0.01 (**). *p* < 0.001 (***).

To characterize the role of TKTL1 for the metabolic response of AML cells to hypoxia, we initially evaluated the effect of the shRNA-mediated silencing of TKTL1 on the expression of TKTL1 and TKT genes by RT-PCR. We obtained a knockdown efficiency of 63.3% ± 0.03 for TKTL1 expression, whereupon TKT expression was not significantly affected (Figure 1A).

Next, we evaluated the impact of TKTL1 knockdown on cell proliferation during hypoxia. At 96 h of cell incubation, an increase in duplication time of TKTL1^KD^ cells compared to THP-1^WT^ of around 25% and 29% for normoxia (O_2_ at 21%) or hypoxia (O_2_ at 1%), respectively, was observed (Table 1). When compared to normoxia, hypoxia induced a 3-fold increase in duplication time of both THP-1^WT^ and THP-1^KD^ cells. These results reinforce the notion that TKTL1 is a necessary enzyme for proliferation, as reported in many previous studies, highlighting that its function is essential under normal oxygen and hypoxic conditions.

Since it is thought that the synthesis of R5P and other pentoses formed in both branches of the PPP mainly contribute to proliferation, we determined the effect of TKTL1 knockdown on the enzyme activity of TKT, G6PD and 6-phosphogluconate dehydrogenase (6PGD) using spectrophotometric methods (see Section 4.3). TKTL1 knockdown resulted in a decrease in transketolase activity of around 45% in normoxia and 49% in hypoxia (Figure 1B) and a decrease in G6PD activity of around 65% and 75% for normoxia and hypoxia, respectively (Figure 1C). By contrast, the knockdown did not alter 6PGD activity in normoxia nor in hypoxia (Figure 1D).

Through transcriptomic analysis, we verified the following differences in activity originating from differential expression. The THP-1^WT^ and THP-1^KD^ changes in gene expression levels in response to hypoxia are listed in Table A1 and Table A2, respectively, in Appendix A.

**Figure 2:**
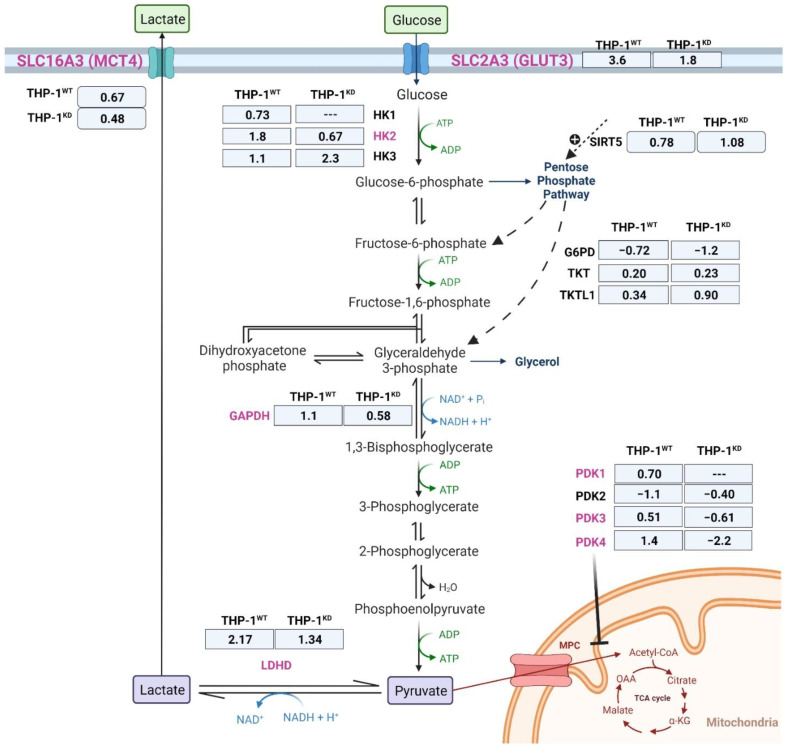
Gene expression changes of key enzymes and transporters between THP-1^WT^ and THP-1^KD^ in hypoxia. Significantly changing genes due to TKTL1 highlighted in pink. Expression changes in normoxia versus hypoxia, indicated in log2-fold-change of n = 3 samples per condition. Abbreviations: G6PD—glucose-6-phosphate dehydrogenase, GAPDH—glyceraldehyde-3-phosphate dehydrogenase, GLUT3—glucose transporter 3, HK1–3—hexokinase 1 to 3, LDHD—lactate dehydrogenase D, MCT4—monocarboxylate transporter 4, PDK1–4—pyruvate dehydrogenase kinase 1 to 4, SIRT5—sirtuin 5, SLC—solute carrier family, TKT—transketolase, TKTL1—transketolase-like 1. Created using BioRender.com (accessed date: 31 January 2022).

Consistent with the hypoxia-induced decrease in proliferation rates of these cells, we observed that G6PD expression in hypoxia (quantified as Log2-fold-change in hypoxia versus normoxia transcript levels (Log2FC)) was decreased significantly for THP-1^WT^ (Log2FC = −0.72; padj < 0.05) as well as for TKTL1^KD^ (Log2FC = −1.24; padj < 0.05) (Figure 2). Considering that G6PD is regulated by post-translational modifications (PTMs), the fact that G6PD activity did not change could be due to PTM enzymatic activation. In fact, it has been described that G6PD is activated by SIRT5-catalyzed deglutarylation [19]. The transcriptomic results show that under hypoxic conditions, SIRT5 levels increased in both THP-1^WT^ (Log2FC = 0.78; padj < 0.05) and THP-1^KD^ (Log2FC = 1.08; padj < 0.05) (Figure 2). With respect to the TKT and TKTL1 genes, there was a very slight increase in gene expression induced by hypoxia, yet with no change in transketolase activity (see Figure 1B).

### 2.2. TKTL1 Is Essential to Trigger Hypoxia-Induced Changes for the Fate of Glucose in THP-1 Cells

**Figure 3:**
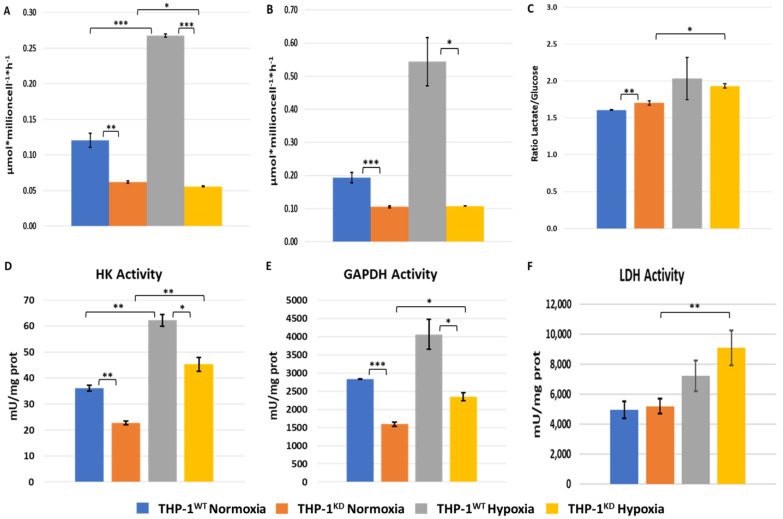
Loss of TKTL1 impairs hypoxia response. Changes in concentration of glucose (**A**) and lactate (**B**) in incubation medium, normalized by cell number and incubation time. Glucose and lactate concentrations were measured in incubation medium of THP-1^WT^ and THP-1^KD^ cells incubated for 48 h, in both normoxic and hypoxic conditions. (**C**) Ratio of change in lactate per change in glucose concentration. Enzymatic activity assays performed through spectrophotometry in normoxia and hypoxia for enzymes (**D**) hexokinase (HK), (**E**) glyceraldehyde-3-phosphate dehydrogenase (GAPDH) and (**F**) lactate dehydrogenase (LDH). Data represented are mean ± SD, with n = 3 in panels A, B and C, and n = 2 in panels D, E and F. Statistically significant differences in all panels were determined by two-tailed independent sample Student’s *t*-test: *p* < 0.05 (*). *p* < 0.01 (**). *p* < 0.001 (***).

A common behavior of most cancer cells under hypoxic conditions is the increase in the glycolytic pathway flux as a metabolic adaptation to their environment. To see how this feature is dependent on TKTL1, we measured the changes in glucose and lactate concentrations in cell culture media of THP-1^WT^ and THP-1^KD^ cells incubated for 48 h under normoxic and hypoxic conditions. In normoxia, THP-1^WT^ glycolytic parameters (changes in glucose and in lactate concentrations) were around 2-fold higher than in THP-1^KD^ cells (Figure 3A–C). The ratio of increased lactate per decreased glucose concentrations in cell culture media was significantly different in WT vs. KD cells in normoxia but not in hypoxia (Figure 3C). Nevertheless, the increased values of around 2 for both cell lines in hypoxia indicate that under O_2_ restriction, glucose is readily oxidized to lactate.

Hypoxia triggered a metabolic switch, as evidenced by a 2.2-fold increase in the change in glucose and lactate concentrations in the incubation medium of THP-1^WT^ cells, whereas in THP-1^KD^, these glycolytic parameters were not statistically different in hypoxia vs. normoxia (Figure 3A,B). These results unveil a key role of TKTL1 in the adaptive metabolic switch to glycolysis that THP-1^WT^ cells undergo under hypoxia.

It has been well-documented that the induction of hypoxia responses in induction of glycolysis occurs as a consequence of increased gene expression of enzymes and transporters (identified in Figure 2) [20,21,22,23,24,25,26,27,28,29]. We investigated whether the impaired enhancement of glycolysis in response to hypoxia observed in THP-1^KD^ was caused by a failure to upregulate gene expression.

Results showed that the expression of the isoforms of hexokinase that bind to mitochondria (HK1 and HK2) and the pyruvate dehydrogenase kinase isoenzymes (PDK1, 3 and 4) were strongly up-regulated in THP-1^WT^ in response to hypoxia. On the contrary, in THP-1^KD^, hypoxic conditions induced a decrease in the expression of PDK3 and PDK4 but did not alter the expression of HK1 nor PDK1. It is worth noting that the hypoxia-induced increase in gene expression of the hexokinase isoenzyme HK3, that binds to the nucleus, is 2.5-fold higher in THP-1^KD^ than in THP-1^WT^, suggesting a TKTL1-independent mechanism of regulation of the expression of this gene in hypoxia. In fact, HK3 has been described to have a role in decreasing reactive oxygen species (ROS) and to have a perinuclear localization [30].

Other glycolytic enzymes and transporters that are reported to be overexpressed in hypoxia, such as glyceraldehyde-3-phosphate dehydrogenase (GAPDH), glucose transporter SLC2A3 (GLUT3), lactate dehydrogenase D (LDHD) and lactate transporter SLC16A3 (also known as monocarboxylate transporter MCT4) were found to be upregulated under hypoxic conditions in both THP-1^WT^ and to a lesser extent also in THP-1^KD^.

Measurement of total HK and GAPDH activity in THP-1^WT^ and THP-1^KD^ cells (Figure 3D,E) showed that the activity was higher in THP-1^WT^ than in THP-1^KD^ in both normoxic and hypoxic conditions. On the contrary, we observed that the knockdown of TKTL1 did not affect total LDH activity and that its activity was increased in both THP-1^WT^ and THP-1^KD^ cells under hypoxia (Figure 3F). The obtained results are consistent with the lower *K*_m_ for glucose reported for HK3 isoenzyme, with respect to HK1 and HK2 [31]. LDHD has been reported to function as a “metabolite repair enzyme” that controls metabolic damage in glycolysis by contributing to the elimination of methylglyoxal formed from another glycolytic enzyme, namely the triose-phosphate isomerase (TPI) [32]. Total GAPDH activity (Figure 3E) as well as GAPDH gene expression (Figure 2) increased in both cell lines in response to hypoxia, favoring the THP-1^WT^.

Altogether, the gene expression profile observed provides strong evidence that the knockdown of TKTL1 affects the metabolic switch to glycolysis in response to hypoxia through impaired upregulation of gene expression for HK and PDKs isoforms. Our observations demonstrated that hypoxia triggers a dramatic increase in Estrogen-Related Receptor b (ESRRb) (log2FC = 4.6; padj < 0.05) in THP-1^WT^, whereas ESRRb levels remain constant in THP-1^KD^. The family of Estrogen-Related Receptors (ESRRs) has been described to mediate PDKs gene overexpression in hypoxia [26,33], allowing us to hypothesize that TKTL1 plays a key role in the switch from OXPHOS to glycolysis by induction of PDKs that result in pyruvate dehydrogenase (PDH) inhibition in an ESRRb-mediated way.

### 2.3. TKTL1 Is Essential to Trigger Hypoxia-Induced Changes to the Fate of Glutamine in THP-1 Cells

**Figure 4:**
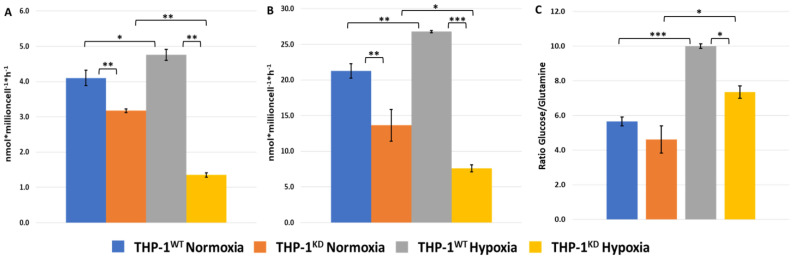
Loss of TKTL1 impairs hypoxia response. Changes in glutamate (**A**) and in glutamine (**B**) concentrations in incubation medium, normalized by cell number and incubation time. Glutamate and glutamine concentrations were measured in incubation medium of THP-1^WT^ and THP-1 ^KD^ cells incubated for 48 h, in both normoxic and hypoxic conditions. (**C**) Ratio of change in glucose per change in glutamine concentration. Data represented are mean ± SD (n = 3). Statistically significant differences in all panels were determined by two-tailed independent sample Student’s *t*-test: *p* < 0.05 (*). *p* < 0.01 (**). *p* < 0.001 (***).

Second to glucose, glutamine is a major carbon source for energy production and anabolic processes [34]. It has been described that hypoxia enhances glutamine synthesis and uptake in cancer cells by increasing glutamine synthetase (GS, also known as GLUL) and the number of glutamine transporters such as SLC38A2 [34]. To attest how TKTL1 could impact glutamine metabolism, we measured the changes in glutamine and glutamate concentrations in incubation medium of THP-1^WT^ and THP-1^KD^ cells incubated for 48 h under normoxic and hypoxic conditions. We found that TKTL1 knockdown resulted in a 20–25% decrease in the change in glutamine and glutamate concentrations under normoxia and in a 70–75% decrease under hypoxia (Figure 4A,B). When comparing the effect of this knockdown on the ratio of change in glucose concentration vs. change in glutamine concentration (Figure 4C), we observed a decrease of around 20% in normoxia and of 27% in hypoxia, which indicates that the overall impact of silencing TKTL1 is more pronounced in hypoxia. In fact, the hypoxic metabolic switch triggered an increase of around 20% for glutamine and glutamate concentrations in THP-1^WT^ cells, while the THP-1^KD^ cells in hypoxia had around a 50–60% decrease in these metabolites’ levels (Figure 4A,B).

**Figure 5:**
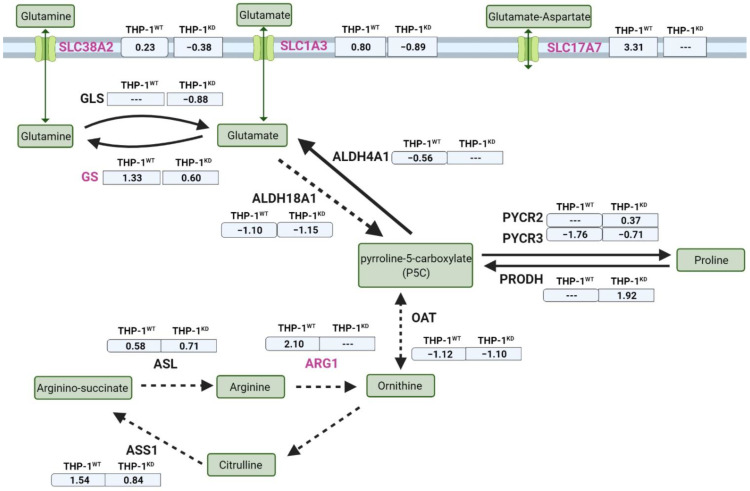
Gene expression alterations of key players in glutamine and glutamate metabolism and transport between THP-1^WT^ and THP-1^KD^ in hypoxia. Significant changing genes due to TKTL1 highlighted in pink. Expression changes in normoxia versus hypoxia, indicated in log2-fold-change for n = 3 samples per condition. Abbreviations: ALDH—aldehyde dehydrogenase family, ALDH4A1—pyrroline-5-carboxylate dehydrogenase. mitochondrial, ALDH18A1—pyrroline-5-carboxylate synthase, ARG1—arginase 1, ASL—argininosuccinate lyase, ASS1—argininosuccinate synthase 1, GLS—glutaminase, GS—glutamine synthetase, OAT—ornithine aminotransferase, PRODH—proline dehydrogenase, PYCR2 and 3—pyrroline-5-carboxylate reductase 2 and 3, SLC—solute carrier family. Created with BioRender.com (accessed on 31 January 2022).

Another key characteristic adaptation to hypoxia in cancer cells is observed in altered expression of key glutamine metabolism genes and transporters [34]. We investigated whether the contrary response seen in hypoxia in our results was due to a failure in the upregulation of gene expression (Figure 5). We observed that GS gene expression was significantly upregulated (log2FC = 1.3; padj < 0.05) and also SLC38A2 was slightly upregulated in THP-1^WT^, whereas glutaminase (GLS) expression did not change. By contrast, GLS and SLC38A2 were downregulated in THP-1^KD^ and the observed overexpression of GS (log2FC = 0.60; padj < 0.05) was 50% lower than that observed in the control cells. In addition, SLC1A3 (glutamate–aspartate transporter) and SLC17A7 (glutamate transporter) were strongly overexpressed under hypoxia conditions in THP-1^WT^, whereas in THP-1^KD^ they were downregulated or unaltered, respectively. Altogether, the observed changes at the transcriptomic level relating to glutamine/glutamate intake and metabolism correlate with an impairment of the metabolic response induced by hypoxia as a consequence of TKTL1 knockdown.

### 2.4. TKTL1 Is Essential to Trigger Hypoxia-Induced Changes in the Fate of Amino Acids in THP-1 Cells

**Figure 6:**
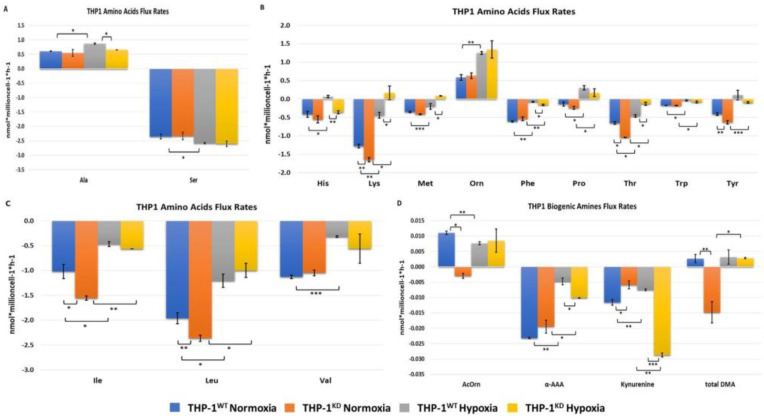
Changes in concentration of specific metabolites measured in incubation medium of THP-1^WT^ and THP-1 ^KD^ cells incubated for 48 h, for both normoxic and hypoxic conditions. All changes in concentrations were normalized by cell number and incubation time. Negative values denote a decrease in concentration during incubation time, and a positive value denotes an increase in concentration during incubation time. (**A**) Alanine and serine. (**B**) Branched-chain amino acids isoleucine (Ile), leucine (Leu) and valine (Val). (**C**) Histidine (His), lysine (Lys), methionine (Met), ornithine (Orn), phenylalanine (Phe), proline (Pro), threonine (Thr), tryptophan (Tryp) and tyrosine (Tyr). (**D**) Acetylornithine (AcOrn), alpha-aminoadipic acid (αAAA), kynurenine and total dimethylarginine (total DMA). Data represented are mean ± SD (n = 3). Statistically significant differences were determined by two-tailed independent sample Student’s *t*-test with *p*-values of *p* < 0.05 (*). *p* < 0.01 (**) and *p* < 0.001 (***).

Besides glutamine and glutamate, we also analyzed and compared the changes in concentrations of amino acids in the incubation medium from THP-1^WT^ and THP-1^KD^ cells incubated in normoxic and hypoxic conditions. Concentrations in the medium of proline and ornithine increased more in THP-1^WT^ than THP-1^KD^ in response to hypoxia (Figure 6B). The observed major increase in gene expression of proline dehydrogenase (PRODH), that converts proline in pyrroline-5-carboxylate (5PC), and of arginase 1 (ARG1), that converts arginine into ornithine, is in accordance with the observed increase in metabolite concentration in incubation medium between THP-1^WT^ and THP-1^KD^ (Figure 5). Moreover, in THP-1^WT^, we observed for hypoxia a lower increase in the concentration of acetylornithine in incubation medium (Figure 6D), the latter being an intermediate metabolite between glutamate and ornithine [35]. This finding is consistent with a more active production of ornithine in the wild-type cells under hypoxic conditions. Interestingly, we also observed a significant increase in the excretion of total dimethylarginines (total DMA) in THP-1^KD^ cells (Figure 6D), which is consistent with a significant overexpression of arginine methyltransferase 8 (PRMT8) (log2FC = 5.7; padj < 0.05). It is worth noting that PRMP8 has been recently described as a key player in maintaining stress tolerance by ensuring proper DMA levels [36].

**Figure 7:**
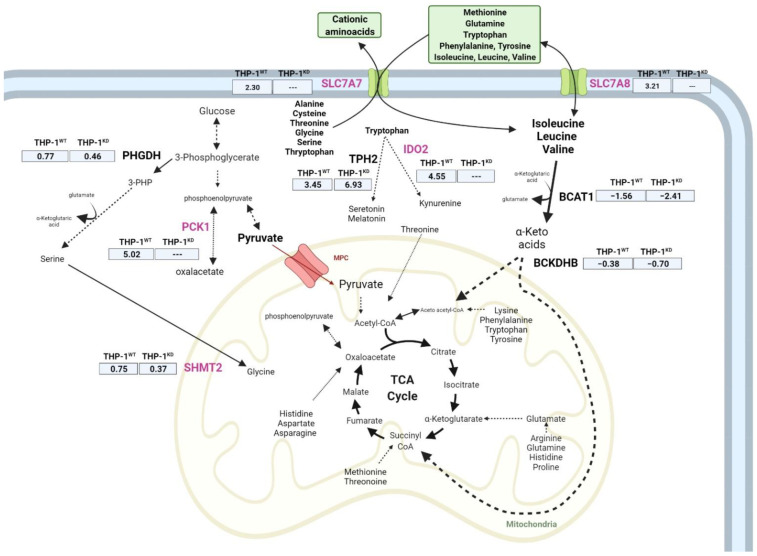
Gene expression changes in amino acid transport and catabolism between THP-1^WT^ and THP-1^KD^ in hypoxia. Significant changing genes due to TKTL1 highlighted in pink. Expression changes in normoxia versus hypoxia, indicated in log2-fold-change of n = 3 samples per condition. Abbreviations: BCAT1—branched-chain amino acid transaminase 1, BCKDHB—branched-chain keto acid dehydrogenase B, IDO2—indoleamine 2,3 dioxygenase 2, PCK1—phosphoenolpyruvate carboxykinase 1, PHGDH—phosphoglycerate dehydrogenase, SHMT2—serine hydroxymethyltransferase 2, SLC—solute carrier family, TPH2—tryptophan hydroxylase 2. Created with BioRender.com (accessed on 31 January 2022).

Regarding branched-chain amino acid (BCAA) metabolism, we observed, for hypoxia, a significantly reduced consumption of isoleucine (Ile) and leucine (Leu), at similar extents, for both THP-1^WT^ and THP-1^KD^ cells. With respect to valine (Val), we observed that the decrease in the change in valine concentration in incubation medium was only statistically significant in THP-1^WT^ cells (Figure 6C). To complete the characterization of BCAA metabolism, we further looked at the gene expression of key enzymes in their catabolism and found that the expression of branched-chain aminotransferase 1 (BCAT1) and branched-chain keto acid dehydrogenase B (BCKDHB) genes decreased under hypoxia in both cell lines. The inhibition on the expression levels of these enzymes was around 1.6-fold higher in THP-1^KD^ than in THP-1^WT^ (Figure 7), which is consistent with the observed higher decrease in Ile and Leu consumption induced by hypoxia in THP-1^KD^.

SLC7A8, an antiport transporter that intakes BCAAs as well as other neutral amino acids (tyrosine, phenylalanine, tryptophan, methionine and glutamine) through obligatory exchange mechanisms, as well as SLC7A7 [37], another antiport transporter that similarly exchanges neutral for cationic amino acids, were dramatically overexpressed in response to hypoxia only in THP-1^WT^ (Figure 7).

Methionine and tyrosine consumption by cells decreased under hypoxia, but the changes were statistically significant only in THP-1^KD^ cells. On the contrary, the decrease in consumption of histidine induced by hypoxia was significant only for THP-1^WT^.

Net lysine, threonine and phenylalanine consumptions decreased significantly under hypoxia in both cell lines, but the changes in concentration in incubation medium observed for lysine and threonine were much larger in THP-1^KD^ (Figure 6B). With respect to phenylalanine, the observed changes were larger in THP-1^WT^ (Figure 6B). It is worth noting that the net consumption of α-aminoadipic acid (αAAA), a biogenic amine generated in the intermediate steps of the catabolism of lysine, decreased under hypoxia in both cell lines (Figure 6D). However, the observed decrease was lower for THP-1^KD^, with cells consuming significantly more αAAA in hypoxia compared to THP-1^WT^. In normoxic conditions, there were no significant differences in αAAA net consumption between the cell lines.

Tryptophan uptake was slightly decreased in both cell lines under hypoxia (Figure 6B). Tryptophan can be metabolized to kynurenine with indoleamine 2.3-dioxygenase 2 (IDO2), the latter being the rate-limiting step of this pathway, or to serotonin through TPH1 and TPH2. Both pathways produce biogenic amines and other active metabolic intermediates with signaling functions. Gene expression of IDO2 was strongly enhanced under hypoxia only in THP-1^WT^, which is consistent with a decreased net consumption of kynurenine in these cells (Figure 6B). On the contrary, kynurenine net consumption was strongly enhanced under hypoxia in THP-1^KD^ cells, which is consistent with the inability of the KD cells to enhance IDO2 in response to hypoxia.

Serine net consumption was also slightly enhanced in hypoxia in both cell lines, but the increase was only statistically significant in THP-1^WT^, in accordance with the observed greater increase in phosphoglycerate dehydrogenase (PHGDH) and serine hydroxymethyltransferase 2 (SHMT2) gene expression in THP-1^WT^ (Figure 7).

Secretion of alanine to the medium was also enhanced under hypoxia, but only for THP-1^WT^ (Figure 6A), which is consistent with the enhanced change in glucose and glutamine concentrations measured in incubation medium of THP-1^WT^ previously observed with respect to THP-1^KD^.

We also measured intracellular amino acid content to see whether the pattern of alterations in THP-1^KD^ incubation medium under hypoxic conditions is consistent with net-total amino acids’ intracellular content. The THP-1^WT^ cells had three times less aspartate and five times more serine in hypoxia, whereas these metabolite levels did not significantly change intracellularly under hypoxia in THP-1^KD^ cells (Table A3 in Appendix B). The observed THP-1^WT^ decrease in aspartate intracellularly under hypoxia is consistent with reported evidence across different tumors of a negative correlation between hypoxia and intracellular aspartate content [38].

The rise in intracellular serine content triggered by hypoxia in THP-1^WT^ together with the previously observed increase in serine net consumption (Figure 6A) could be related to the use of this amino acid for pyrimidine synthesis and cell proliferation under hypoxia. In fact, it has been described that serine synthesis through PHGDH is crucial for the maintenance of nucleotide levels [39], and a similar effect was reported for phosphoenolpyruvate carboxykinase 1 (PCK1), which is a key player that drives pyrimidine biosynthesis under hypoxia [40]. It is worth noting that PCK1 gene expression was significantly increased (log2FC = 5.0; padj < 0.05) under hypoxia in THP-1^WT^, whereas hypoxia did not trigger any statistically significant changes in THP-1^KD^ (Figure 7).

The fact that we did not observe these changes in intracellular amino acid content in hypoxia in THP-1^KD^ corroborates the importance of TKTL1 in triggering the switch to enhanced glycolysis and the consequent adaptation of amino acid metabolism to support such a response. Overall, this new role of TKTL1, beyond its enzymatic function on the non-oxidative branch of the PPP unveiled in this study, opens new opportunities for the design of combined therapies targeting TKTL1 and for exploiting the impaired adaptation of these cells to hypoxic stress.

## 3. Discussion

Leukemia is a type of cancer characterized by a rapid expansion of immature hematopoietic cells [41]. This uncontrolled proliferation can arise from many distinct conjugations of mutations, whose compiled synergy drives tumorigenesis [42,43,44,45,46]. The resulting phenotype presents a different metabolism from the surrounding normal cells, granting it advantages in proliferation and survival in the hypoxic conditions of the hematopoietic niche [47,48,49,50,51].

Transketolase-like 1 is a key enzyme of metabolism, linking glycolysis to the production of nucleotides, a necessary component for de novo synthesis of RNA and DNA, especially in rapidly proliferating cells [52,53,54]. In fact, expression of TKTL1 is low in normal tissues except for testis [55,56]. Although the full scope of its functions is still unknown to this day, a clear correlation of its expression with cancer malignancy and poor prognosis has been established across many types of cancer [5,6,7,57,58]. One such type is AML, where the putative role of TKTL1 overexpression remains to be properly pursued [16,59].

This study combined targeted metabolomics and transcriptomics profiling for a comparative analysis of a stable, acute, monocytic leukemia cell line with a TKTL1 knockdown and a wild-type counterpart, in order to discern TKTL1’s role in metabolic adaptation under the state of hypoxia.

The results obtained unveiled that the main metabolic changes triggered by hypoxic conditions, such as increased glucose and glutamine consumptions and increased lactate production, are impaired by TKTL1 knockdown. These differences occur as a consequence of changes at the level of gene expression, enzyme activities, metabolite exchange with extracellular media and intracellular metabolite homoeostasis.

In fact, we have observed impaired gene expression and/or enzyme activity of GAPDH, a glycolytic protein transcriptionally upregulated in hypoxia with moonlighting activities [60], and other key metabolic players of glucose metabolism reported in the literature to be induced under hypoxia [20,21,22,23,24,25], such as lactate and glucose transporters, PDKs, HKs, PCK1, and LDHs. Moreover, TKTL1 knockdown results in a decrease in G6PD activity, an enzyme whose main function is to maintain redox homeostasis [61], thus expanding the metabolic impact on pentose-phosphate and NADPH synthesis capabilities of the TKTL1-knockdown cells. Worth noting that the impaired increase in GAPDH and G6PD, the key enzymes of the upper part of glycolysis and the oxidative branch of the PPP pathway, can indirectly contribute to the observed decrease in TKT activity to maintain metabolic homeostasis. Moreover, the decrease in reducing power associated with decreases in GAPDH and G6PD, two critical mediators of the cellular response to oxidative stress, will impact every aspect of energetic metabolism.

At the level of glutamine–glutamate transport and metabolism, we also observed that the expression of key genes and transporters, reported in the literature to be induced by hypoxia [34], such as SLC1A3, SLC17A7, SLC38A2 and glutamine synthase, was also impaired in TKTL1 knockdown cells when submitted to the hypoxia challenge.

It is worth noting that loss of TKTL1 also resulted in a reduction in the hypoxia-triggered switch to proline production when compared with wild-type cells, and that the expression pattern of key players in proline metabolism (PYCRs and PRODH) is also altered. Taking into account that TKTL1 knockdown results in a decrease in G6PD activity and that proline synthesis requires NADPH, these results could be explained by a decreased NADPH pool triggered by G6PD activity deficiency. In fact, there is increasing evidence that proline synthesis plays a key role in the regulation of cellular redox homeostasis and that it is synthesized beyond the cell proliferative need as part of a “redox valve” mechanism [62].

Another consequence of impaired GAPDH activity induced by TKTL1 is the possible effects in serine synthesis. It has been described that GAPDH controls D-serine synthesis in astrocytes [63]. The impaired expression in hypoxia of GAPDH, together with PCK1 and key cationic and essential amino acids transporters (SLC7A7 and SLC7A8), as well as of the attenuated expression of key players in serine synthesis, could be related to an altered pyrimidine nucleotide synthesis owing to TKTL1 knockdown. In fact, it has been described that PCK1 and PHGDH are key players in maintaining appropriate nucleotide synthesis under hypoxia [39,40].

The fact that TKTL1 knockdown results in downregulation of so many metabolic genes indicates that TKTL1 could be a “moonlight protein” and may well also act by other mechanisms not involving enzyme activity. Further studies are required to investigate whether TKTL1 catalyzes a mono-substrate reaction or an unsuspected reaction that could induce transcription factors.

Of note, the cells in hypoxia initiated a compensatory mechanism in order to survive in response to TKTL1 knockdown. Increased gene expression of SIRT5 stimulates the regulation of G6PD through post-translational modifications in order to maintain its activity in hypoxia. Increased use of the isoenzyme HK3 and upregulation of PRMT8 are clear responses to ROS increase, since both have been reported to mediate mitochondrial biogenesis and stress tolerance.

While there is previous evidence demonstrating that TKTL1 expression is triggered in hypoxia [13,14], our study goes beyond the state of the art, unveiling the metabolic role of TKTL1 triggering the switch to enhanced glycolysis and glutamine consumption at the molecular level. In fact, our results provide evidence that TKTL1 plays a key role in facilitating the overexpression of key proteins necessary for the switch to enhanced glycolysis and glutamine consumption modes characteristic of hypoxia adaptation.

Therefore, our results reveal that the role of TKTL1 in the adaptation to hypoxia is essential for the coordination of central metabolism to trigger metabolic reprogramming, both regulating glucose/glutamine consumption and also contributing to oxidative stress regulation through the overexpression of GAPDH and G6PD.

Further studies should be carried out to better understand the mechanisms involved in this new facet of TKTL1 function in metabolism. Additional investigations into the changes identified here on other AML cell lines and primary patient-derived cells could reveal new approaches for therapies that combine targeting TKTL1 and the “weaknesses” that arise from its knockdown.

## 4. Materials and Methods

### 4.1. Cell Culture

The THP-1 cell line is a human monocytic cell line derived from an acute monocytic leukemia patient, and is widely used as a model for AML in drug screenings. THP-1 cell line was obtained from the American Type Culture Collection (Manassas, VA, USA). THP-1^WT^ and THP-1^KD^ cells were generated by Sirion Biotech GmbH (Munich, Germany). THP-1 cells were grown in suspension in RPMI-1640 culture medium of 10 mM glucose, 2 mM glutamine and supplemented with 10% heat-inactivated fetal calf serum (FCS) and 1% penicillin/streptomycin at 37 °C and 5% CO_2_. In hypoxia settings, cells were cultured for 5 days for chronic hypoxic adaptation in Hypoxystation H35 (Don Whitley Scientific Limited, Bingley, UK) prior to any experiment.

### 4.2. Cell Viability Assays

Cell proliferation curves were performed by cell counting using a Scepter^TM^ Handheld Automated Cell Counter (Merck Millipore, Burlington, MA, USA) and Countess II Automated Cell Counter (Thermo Fisher Scientific, Waltham, MA, USA). THP-1 cell lines were seeded at 3 × 10^5^ cells per 25 cm^2^ flasks in normoxia and at 4 × 10^5^ cells in hypoxia. Cell counting was performed at 24 h timepoints up until a total of 96 h. After counting, THP-1 cells were collected in Eppendorf tubes from seeding flasks, with volumes of 500 μL, centrifuged at 350× *g* for 5 min, supernatant was removed and resuspended in 1 mL PBS solution (phosphate-buffered saline).

### 4.3. Measurement of Enzyme Activities, Concentration of Metabolites in Incubation Medium, and Intracellular Concentrations of Glucose, Lactate, Amino Acids, and Biogenic Amines

Specific enzyme activities of G6PD, GAPDH, HK, LDH, PK, 6PGD and TKT were determined by using NAD(P)H-coupled enzymatic reactions [64].

For measurement of consumption and production rates of metabolites and measurement of intracellular concentrations, THP-1 cell lines were seeded at 4 × 10^5^ cells/mL for both normoxia and hypoxia. After 48 h incubation, extracts were collected from 5 × 10^6^ cells each. Media and cell pellets were frozen until analysis.

Changes in metabolite concentrations in incubation media were determined by measuring metabolite concentration at the beginning and end of incubation time and normalizing the difference between these two concentrations by time and cell number. Exponential cell growth was considered during the entire incubation time [65]. Glucose, lactate, glutamate and glutamine concentrations in incubation media aliquots were measured using NAD(P)H-coupled enzymatic reactions in a COBAS Mira Plus spectrophotometer (Horiba ABX, Kyoto, Japan) [64].

The concentration of amino acids and biogenic amines in incubation media was determined using the AbsoluteIDQ™ p180 Kit (Biocrates Life Sciences, Innsbruck, Austria) and the AB Sciex 4000 QTRAP MS/MS mass spectrometer coupled to an Agilent HPLC 1200, according to manufacturer’s instructions. Next, 10 μL of media were plated in each well of the kit. Analyst and the MetIDQ™ software packages were used to analyze the obtained data and calculate metabolite concentrations.

Amino acids’ and biogenic amines’ intracellular concentrations were determined from cell lysates using the AbsoluteIDQ™ p180 Kit (Biocrates Life Sciences, Innsbruck, Austria) and the AB Sciex 4000 QTRAP MS/MS mass spectrometer coupled to an Agilent HPLC 1200 (AB Sciex LLC, Framingham, MA, USA; Agilent Technologies, Santa Clara, CA, USA). Cell pellets containing ca. 5 × 10^6^ cells were resuspended in 70 μL of EtOH:PBS 85:15. Suspensions were treated twice as follows: suspensions were sonicated using titanium probe (3 × 15 s; output 25, tune 50), then submerged in liquid N_2_ for 30 s and thawed at 95 °C in a dry bath. Subsequently, suspensions were centrifuged at 20,000× *g* for 5 min at 4 °C, and supernatants were collected. Then, 30–50 μL of supernatant was plated in each well of the kit. Analyst and the MetIDQ™ software packages were used to analyze the obtained data and calculate metabolite concentrations. Intracellular concentrations were corrected by protein content in cell lysates, measured using bicinchoninic acid (BCA) assay.

### 4.4. Real-Time PCR

RNA was extracted from THP-1 cells, both fresh and frozen extracts using Trizol (Sigma-Aldrich Co LLC, Saint Louis, MO, USA) according to the manufacturer’s protocol. Chloroform was added to the mixture and centrifuged to generate aqueous and organic phases. The aqueous phase was collected, added to cold isopropanol and incubated overnight at 4 °C to precipitate the RNA. Samples were then centrifuged at 14,000× *g* at 4 °C for 15 min. RNA was purified with several washing steps using 75% ethanol and resuspended in RNAse-free water. Purified RNA was quantified using Nanodrop Spectrophotometer (ND 1000 V3.1.0. Thermo Fisher Scientific Inc., Waltham, MA, USA).

The Reverse Transcription reaction (converting RNA into DNA) was performed at 37 °C using 1 μg of RNA added to a mixture containing 5× Buffer (Invitrogen, Waltham, MA, USA), 0.1 M dithiothreitol (DTT) (Invitrogen, Waltham, MA, USA), Random Hexamers (Roche, Basilea, Switzerland), 40 U·μL^−1^ RNAse inhibitor (Promega, Fitchburg, WI, USA), 40 mM dNTPs (Bioline, London, UK), 200 U·μL^−1^ M-MLV-RT (Invitrogen, Waltham, MA, USA).

Gene-expression analysis was performed with a RT-PCR system (Applied Biosystems^®^ 7500 Real Time PCR, Applied Biosystems, Waltham, MA, USA) using the manufacturer’s standard protocol employing TaqMan^®^ (Applied Biosystems, Waltham, MA, USA) and gene-specific probes for TKT (Hs00169074_m1), TKTL1 (Hs00202061_m1), GLS (Hs01014019_m1). Reactions were performed in a volume of 20 μL containing 9 μL of cDNA and 11 μL of TaqMan Master Mix (Applied Biosystems, Waltham, MA, USA). RT-PCR program set-up parameters were: (1) initial incubation at 50 °C for 2 min, (2) denaturalization at 95 °C for 10 min, (3) amplification of 40 cycles alternating between 95 °C for 15 s and 60 °C for 1 min. The house-keeping gene used as reference was PP1A (Hs99999904_m1, Applied Biosystems) and expression levels were quantified using the ΔΔCt method.

### 4.5. Transcriptomics

#### 4.5.1. RNA Extraction

Cell pellets of THP-1 cell lines were collected and frozen. Total RNA from lysates of the THP1^WT^ and THP1^KD^ cell lines in triplicates (n = 3) under normoxic and hypoxic conditions, and with 10 × 10^6^ cells each, were extracted using the RNeasy Mini Kit (Qiagen, Hilden, Germany). RNA integrity was further tested using lab-on-a-chip technology on the BioAnalyzer 2100.

#### 4.5.2. RNA-Seq Library Preparation, Sequencing and Generation of FastQ Files

High-quality RNA-seq (transcriptome) was performed in the CNIC (National Centre for Cardiovascular Diseases, Madrid, Spain) genomic unit using the Illumina HiSeq 2500 sequencer. There, 200 ng of total RNA was used to generate barcoded RNA-seq libraries using the NEBNext Ultra RNA Library preparation kit (New England Biolabs, Ipswich, MA, USA). Briefly, poly A+ RNA was purified using poly-T oligo-attached magnetic beads followed by fragmentation and then first and second cDNA strand synthesis. Next, cDNA ends were repaired and adenylated. The NEBNext adaptor was then ligated, followed by uracil excision from the adaptor and PCR amplification. Finally, the size of the libraries was checked using the Agilent 2100 Bioanalyzer DNA 1000 chip and their concentration was determined using the Qubit^®^ fluorometer (Life Technologies, Carlsbad, CA, USA). Libraries were sequenced on a HiSeq2500 (Illumina, San Diego, CA, USA) to generate 61-base single reads. Finally, FastQ files for each sample were obtained using CASAVA v1.8 software (Illumina, San Diego, CA, USA).

#### 4.5.3. Analysis of the Raw RNA Sequencing Data

The quality of the raw RNA sequencing reads was assessed by FastQC version 0.11.3, a computational quality-control tool for high-throughput sequence data in Java developed by Simon Andrews and the Babraham Bioinformatics group (http://www.bioinformatics.babraham.ac.uk/projects/fastqc/ accessed on 31 January 2022, BaBraham Institute, Cambridge, UK). The alignment of the reads was performed using STAR version 2.5.2a open-source software and the FASTA sequences were generated using Homo sapiens high-coverage GRCh37.75.dna.primary_assembly. Gene-level count tables were obtained using the count script of the HTSeq python library version 0.6.1p1 [66] with default options. Only reads with unique mappings were considered. For performing dimensionality reduction by principal component analysis (PCA) and hierarchical clustering, normalized transcript counts were utilized after transformation with the “rlog” function of the Bioconductor package DESeq2 version 1.12.4 [67]. R version 3.3.1 [68] was used for conducting biostatistical analyses.

#### 4.5.4. Differential Expression Analysis

The statistical analysis to identify differentially expressed genes was performed using the Bioconductor package DESeq2 version 1.12.4 [67]. Size-factor-based normalization was performed to control for batch effects and inter-sample variability. Genes with less than 10 counts across all samples were filtered out. Differential expression analysis was performed using package defaults for dispersion estimation and the function “Deseq”, to cover independent filtering, cooks cutoff [69] for outlier detection and a Wald test. Adjusted *p*-values (padj) were computed from the DESeq2-calculated *p*-values by applying a Bonferroni correction for multiple testing. Genes with a Padj-value < 0.05 were considered as statistically significantly differentially expressed. All aforementioned biostatistical analyses were performed using R version 3.3.1 [68].

### 4.6. Statistical Analysis

Experiments were performed in triplicate for cell lines in at least three independent experiments. Statistical analyses were performed using the parametric unpaired, two-tailed independent sample Student’s *t*-test, assuming normality of distribution and homogeneity of variance based on past experience in similar measurements. *p* < 0.05 (*) was considered statistically significant.

## Figures and Tables

**Table 1:** Duplication times of THP-1^WT^ and THP-1^KD^ under normoxia and hypoxia.

	Duplication Time (hours)	% Increment Versus WT
**THP-1^WT^ Normoxia**	38.4 ± 2.24	--
**THP-1^KD^ Normoxia**	49.4 ± 2.48 **	25.1
**THP-1^WT^ Hypoxia**	113.5 ± 41.81	--
**THP-1^KD^ Hypoxia**	151.3 ± 19.38	28.6

Data represented as mean ± SD (n = 3). Statistically significant differences between THP-1^WT^ and THP-1^KD^ in normoxia or in hypoxia were determined by two-tailed independent sample Student’s *t*-test: *p* < 0.01 (**).

## Appendix A

**Table A1:** Differential gene expression comparing THP1^WT^ cells in hypoxia (n = 3) versus normoxia (n = 3).

Gene Symbol	Description	Log2 Fold Change	lfcSE ^1^	*p*-Value	*p*-adj. ^2^
G6PD	glucose-6-phosphate dehydrogenase	−0.72	0.09	1.45 × 10^−15^	1.41 × 10^−14^
SIRT5	sirtuin 5	0.78	1.10	7.01 × 10^−13^	5.32 × 10^−12^
TKTL1	transketolase-like 1	0.34	0.11	2.4 × 10^−3^	5.8 × 10^−3^
TKT	transketolase	0.20	0.07	2.7 × 10^−3^	6.5 × 10^−3^
HK1	hexokinase 1	0.73	0.12	1.49 × 10^−9^	8.00 × 10^−9^
HK2	hexokinase 2	1.82	0.17	2.71 × 10^−27^	6.56 × 10^−26^
HK3	hexokinase 3	1.09	0.17	2.27 × 10^−10^	1.32 × 10^−9^
PDK1	pyruvate dehydrogenase kinase 1	0.70	0.21	7.6 × 10^−4^	2.0 × 10^−3^
PDK3	pyruvate dehydrogenase kinase 3	0.51	0.21	1.6 × 10^−2^	3.1 × 10^−2^
PDK4	pyruvate dehydrogenase kinase 4	1.42	0.49	4.0 × 10^−3^	9.1 × 10^−3^
GAPDH	glyceraldehyde-3-phosphate dehydrogenase	1.05	0.11	9.71 × 10^−22^	1.59 × 10^−20^
LDHD	lactate dehydrogenase D	2.18	0.25	7.55 × 10^−18^	8.89 × 10^−17^
SLC2A3	solute carrier family 2 member 3	0.67	0.32	3.11 × 10^−8^	1.44 × 10^−7^
SLC16A3	solute carrier family 16 member 3	3.60	0.12	6.36 × 10^−30^	1.81 × 10^−28^
ESRRB	estrogen related receptor beta	4.64	1.4	1.2 × 10^−3^	3.0 × 10^−3^
GLUL	glutamate-ammonia ligase	1.38	0.12	2.16 × 10^−30^	6.37 × 10^−29^
SLC1A3	solute carrier family 1 member 3	0.80	0.29	5.9 × 10^−3^	1.3 × 10^−2^
SLC17A7	solute carrier family 17 member 7	3.31	0.61	5.31 × 10^−8^	2.40 × 10^−7^
SLC38A2	solute carrier family 38 member 2	0.28	0.10	2.6 × 10^−2^	4.9 × 10^−2^
IDO2	indoleamine 2,3-dioxygenase 2	4.55	1.75	9.4 × 10^−3^	2.0 × 10^−2^
PHGDH	phosphoglycerate dehydrogenase	0.77	0.09	3.11 × 10^−19^	4.15 × 10^−18^
SHMT2	serine hydroxymethyltransferase 2	0.75	0.04	7.34 × 10^−65^	1.46 × 10^−6^
PCK1	phosphoenolpyruvate carboxykinase 1	5.03	1.51	8.8 × 10^−4^	2.3 × 10^−3^
TPH2	tryptophan hydroxylase 2	--	--	--	--
BCAT1	Branched chain amino acid transaminase 1	−1.56	0.14	9.14 × 10^−31^	2.74 × 10^−29^
BCKDH	Branched chain keto acid dehydrogenase E1 subunit beta	−0.38	0.10	2.3 × 10^−4^	6.7 × 10^−4^
SLC7A7	solute carrier family 7 member 7	2.28	0.16	6.47 × 10^−46^	4.86 × 10^−44^
SLC7A8	solute carrier family 7 member 8	3.21	0.85	0.00015	0.00044

^1^ lfcSE is the log fold change Standard Error, ^2^ *p*-adj. is adjusted *p*-value.

**Table A2:** Differential gene expression comparing THP1^KD^ cells in hypoxia (n = 3) versus normoxia (n = 3).

Gene Symbol	Description	Log2 Fold Change	lfcSE ^1^	*p*-Value	*p*-adj. ^2^
G6PD	glucose-6-phosphate dehydrogenase	−1.24	0.10	3.15 × 10^−37^	1.51 × 10^−35^
SIRT5	sirtuin 5	1.08	0.12	2.92 × 10^−19^	3.50 × 10^−18^
TKTL1	transketolase-like 1	0.90	0.12	2.14 × 10^−14^	1.66 × 10^−13^
TKT	transketolase	0.24	0.07	5.9 × 10^−4^	1.5 × 10^−3^
HK2	hexokinase 2	0.67	0.17	7.82 × 10^−5^	2.2 × 10^−4^
HK3	hexokinase 3	2.73	0.17	7.21 × 10^−57^	1.17 × 10^−54^
PDK3	pyruvate dehydrogenase kinase 3	−0.61	0.21	3.9 × 10^−3^	8.9 × 10^−3^
PDK4	pyruvate dehydrogenase kinase 4	−2.20	0.53	3.53 × 10^−5^	1.1 × 10^−4^
GAPDH	glyceraldehyde-3-phosphate dehydrogenase	0.57	0.11	1.86 × 10^−7^	7.30 × 10^−7^
LDHD	lactate dehydrogenase D	1.34	0.14	1.53 × 10^−20^	2.03 × 10^−19^
SLC2A3	solute carrier family 2 member 3	1.81	0.32	2.11 × 10^−8^	9.12 × 10^−8^
SLC16A3	solute carrier family 16 member 3	0.48	0.12	7.73 × 10^−5^	2.2 × 10^−4^
ESRRB	estrogen related receptor beta	--	--	--	--
GLUL	glutamate-ammonia ligase	0.60	0.12	2.07 × 10^−7^	8.09 × 10^−7^
GLS	glutaminase	−0.88	0.13	1.67 × 10^−11^	9.95 × 10^−11^
SLC1A3	solute carrier family 1 member 3	−0.89	0.32	5.4 × 10^−3^	1.1 × 10^−2^
SLC38A2	solute carrier family 38 member 2	−0.38	0.10	2.8 × 10^−4^	7.1 × 10^−4^
IDO2	indoleamine 2,3-dioxygenase 2	--	--	--	--
PHGDH	phosphoglycerate dehydrogenase	0.46	0.09	1.10 × 10^−7^	4.42 × 10^−7^
SHMT2	serine hydroxymethyltransferase 2	0.37	0.05	1.81 × 10^−15^	1.53 × 10^−14^
TPH2	tryptophan hydroxylase 2	6.93	1.40	6.88 × 10^−7^	2.53 × 10^−6^
BCAT1	branched chain amino acid transaminase 1	−2.41	0.14	9.87 × 10^−66^	2.61 × 10^−63^

^1^ lfcSE is the log fold change Standard Error, ^2^ *p*-adj. is adjusted *p*-value.

## Appendix B

**Table A3:** Intracellular concentrations of metabolites in THP-1^WT^ and THP-1^KD^ cell lines.

Metabolite	THP-1^WT^ Normoxia	THP-1^KD^ Normoxia	THP-1^WT^ Hypoxia	THP-1^KD^ Hypoxia	*p*-Value THP-1^WT^ (Nor vs. Hyp)	*p*-Value THP-1^KD^ (Nor vs. Hyp)
Alanine	402.30 ± 143.45	473.60 ± 144.63	346.52 ± 5.56	279.01 ± 79.01	0.5378	0.1103
Arginine	273.40 ± 77.02	272.04 ± 150.85	292.70 ± 144.44	111.34 ± 86.22	0.8482	0.1844
Asparagine	771.52 ± 228.94	563.26 ± 54.48	474.11 ± 39.58	702.79 ± 395.83	0.0909	0.5779
Aspartate	118.19 ± 37.52	136.50 ± 46.37	36.98 ± 10.07	55.27 ± 44.06	0.0223	0.0927
Citrulline	0.24 ± 0.23	3.20 ± 4.66	2.54 ± 2.93	0.52 ± 0.89	0.2464	0.3824
Glutamine	1636.46 ± 205.69	1760.44 ± 510.50	1377.17 ± 295.79	1100.21 ± 204.71	0.2806	0.1061
Glutamate	855.50 ± 1481.77	1089.97 ± 943.94	0.00 ± 0.00	1609.70 ± 571.89	0.3739	0.4605
Glycine	915.60 ± 375.13	1075.90 ± 575.77	583.59 ± 285.89	778.96 ± 222.69	0.1685	0.4516
Histidine	72.78 ± 13.73	46.93 ± 17.33	58.96 ± 8.22	36.64 ± 16.40	0.2093	0.4967
Isoleucine	185.40 ± 38.58	200.03 ± 38.97	210.65 ± 76.16	153.94 ± 44.02	0.6355	0.2461
Leucine	236.18± 55.20	248.53 ± 52.65	249.76 ± 63.02	168.81 ± 66.49	0.7928	0.1789
Lysine	40.25 ± 17.59	53.42 ± 8.72	63.64 ± 23.21	38.99 ± 25.69	0.2368	0.4090
Methionine	71.61 ± 20.62	66.44 ± 25.40	63.10 ± 21.25	41.63 ± 22.51	0.6446	0.2741
Ornithine	22.30 ± 10.41	21.32 ± 9.00	35.94 ± 6.38	29.14 ± 16.43	0.1253	0.5096
Phenylalanine	42.47 ± 7.37	39.58 ± 11.15	59.42 ± 22.21	34.28 ± 9.91	0.2781	0.5709
Proline	705.55 ± 139.47	743.98 ± 116.06	249.71 ± 37.70	191.38 ± 38.52	0.0055	0.0014
Serine	70.15 ± 23.94	129.16 ± 90.81	353.88 ± 116.37	186.89 ± 34.27	0.0144	0.3612
Threonine	173.83 ± 92.88	142.23 ± 28.16	200.71 ± 89.78	111.91 ± 40.27	0.7368	0.3455
Tryptophan	11.02 ± 3.72	12.83 ± 1.75	16.12 ± 6.45	9.69 ± 1.04	0.3014	0.0555
Tyrosine	76.14 ± 26.81	91.19 ± 14.14	89.76 ± 32.79	82.07 ± 19.26	0.6074	0.5448
Valine	54.40 ± 19.08	40.16 ± 5.70	60.33 ± 7.25	51.91 ± 20.82	0.6410	0.3992
Acetylornithine	2.44 ± 2.34	1.18 ± 1.91	3.27 ± 1.82	2.57 ± 3.02	0.6537	0.5368
ADMA	2.17 ± 1.48	1.57 ± 0.75	1.31 ± 0.38	0.74 ± 0.69	0.3849	0.2322
α-AAA	7.09 ± 2.44	6.27 ± 2.80	5.35 ± 5.40	4.62 ± 0.51	0.6387	0.3711
Carnosine	0.84 ± 0.55	0.51 ± 0.19	0.38 ± 0.17	0.21 ± 0.09	0.2395	0.0732
Creatinine	16.70 ± 6.86	15.20 ± 2.31	10.38 ± 2.29	11.00 ± 1.91	0.2047	0.0722
Histamine	0.32 ± 0.29	0.23 ± 0.40	0.00 ± 0.00	0.25 ± 0.44	0.1252	0.9480
Kynurenine	0.38 ± 0.17	0.43 ± 0.09	0.39 ± 0.17	0.45 ± 0.16	0.9685	0.9024
Met-SO	1.88 ± 0.58	1.98 ± 0.95	2.81 ± 1.47	2.79 ± 1.40	0.3630	0.4562
Putrescine	2.96 ± 1.32	3.59 ± 1.84	3.00 ± 0.89	3.22 ± 0.51	0.9657	0.7542
Spermidine	45.35 ± 16.23	36.51 ± 5.33	23.81 ± 9.00	26.50 ± 7.43	0.1148	0.1306
Spermine	20.86 ± 11.70	14.61 ± 2.37	13.77 ± 7.30	9.65 ± 4.51	0.4232	0.1667
T4-OH-Pro	221.31 ± 22.58	202.10 ± 40.27	130.55 ± 4.57	130.59 ± 58.46	0.0024	0.1559
Taurine	469.08 ± 82.46	464.12 ± 361.99	196.01 ± 188.34	219.15 ± 200.75	0.0829	0.3633
Total DMA	0.12 ± 0.12	0.05 ± 0.05	0.12 ± 0.12	0.20 ± 0.16	0.9458	0.2015

The intracellular content of these metabolites was obtained using the Biocrates Absolute IDQ^TM^ p180 kit (Biocrates Life Sciences AG, Austria) after 48 h incubation with RPMI 1640 10 mM Glc and 4 mM Gln, 5% S/P and 1% FBS, in both normoxia and hypoxia conditions. Data is represented as mean ± SD (n = 3).
